# α-Conotoxin TxIB: A Uniquely Selective Ligand for α6/α3β2β3 Nicotinic Acetylcholine Receptor Attenuates Nicotine-Induced Conditioned Place Preference in Mice

**DOI:** 10.3390/md17090490

**Published:** 2019-08-22

**Authors:** Shen You, Xiaodan Li, Jian Xiong, Xiaoyu Zhu, Dongting Zhangsun, Xiaopeng Zhu, Sulan Luo

**Affiliations:** Key Laboratory of Tropical Biological Resources of Ministry of Education, Key Laboratory for Marine Drugs of Haikou, School of Life and Pharmaceutical Sciences, Hainan University, Haikou 570228, China

**Keywords:** nicotine addiction, nAChRs, conditioned place preference (CPP), neurotransmitters

## Abstract

α-Conotoxin TxIB is a specific antagonist of α6/α3β2β3(α6β2*) nicotinic acetylcholine receptor (nAChR) with an IC_50_ of 28 nM. Previous studies have shown that α6β2* nAChRs are abundantly expressed in midbrain dopaminergic neurons and play an important role in mediating the mechanism of nicotine and other drugs reward effect. It provided important targets for the development of anti-addiction drugs. The present study evaluated the pharmacological activity of TxIB in vivo with conditioned place preference (CPP) model, which were induced by subcutaneous injection (s.c.) of nicotine (NIC, 0.5 mg/kg). α-Conotoxin TxIB inhibited the expression and reinstatement of CPP in mice dose-dependently, but had no significant effect on locomotor activity. The concentrations of dopamine (DA), γ-aminobutyric acid (GABA) and noradrenaline (NE) in different brain regions were measured by enzyme-linked immunosorbent assay (ELISA). We found that TxIB could inhibit the concentrations of DA, GABA and NE in different brain regions (such as nucleus accumbens (NAc), hippocampus (HIP) and prefrontal cortex (PFC)) in NIC-induced mice. The concentrations of DA and NE were decreased in ventral tegmental area (VTA), while GABA had little change. The current work described the inhibition activity of TxIB in NIC-induced CPP, suggesting that α6β2* nAChR-targeted compound may be a promising drug for nicotine addiction treatment.

## 1. Introduction

Six million people die (including 480,000 people in the US) from diseases that were induced by smoking every year in the world [1]. Smoking is extremely harmful to the human body, especially the respiratory tract, which can easily cause coughing diseases such as laryngitis, bronchitis and emphysema. Nicotine, as the main active ingredient in tobacco, can cause tobacco dependence, because it binds to nicotinic acetylcholine receptors (nAChRs) in the central and peripheral nervous systems [2].

The nAChRs are the superfamily of pentameric ligand-gated ion channels, which are involved in a variety of diseases including nicotine addiction, Alzheimer’s and Parkinson’s disease, schizophrenia and attention deficit hyperactivity disorder, etc. [3,4,5]. It is reported that α6β2* nAChRs (where * denotes possible assembly with other nicotinic receptor subunits) were expressed in dopaminergic neurons of the central nervous system (CNS), such as the nucleus accumbens (NAc) and ventral tegmental area (VTA). It suggested that α6β2* nAChRs may play a key role in regulating emotions and nicotine rewards [6,7]. The α6β2* nΑChRs are also expressed in the catecholamine nucleus of the midbrain region, which is thought to mediate drug reward and enhancement effect in rodents and plays a major role in presynaptic dopamine (DA) release [8]. This makes α6β2* nAChR a potential therapeutic target for the treatment of several neuropsychiatric disorders, including nicotine addiction and Parkinson’s disease [9,10]. In addition, several studies proved that a knock-out of the β2 subunit eliminated nicotine self-administration in mice [11,12].

The venom of conical snails contains up to 100,000 different pharmacologically active compounds worldwide [13,14,15]. These conotoxins specifically target different ion channels, such as Na^+^, K^+^ and Ca^2+^ channels and membrane receptors, such as nAChRs, 5-HT_3_R, NMDAR and G-protein-coupled receptors [16]. According to the number of cysteine residues, the arrangement of disulfide bonds and the consensus signal sequence, conotoxin can be divided into different superfamilies (A, B, C, D, E, I, M, O, P, S, T, etc.) [17]. α-Conotoxin is the first family to be discovered and characteristically targets nAChRs. α-Conotoxin TxIB blocked α6/α3β2β3 nAChR with an IC_50_ of 28 nM, which was tested on rat nAChR heterologously expressed in *Xenopus* oocytes [18]. The designation α6/α3β2β3 nAChR indicates a chimeric receptor which consists of amino acids 1 to 237 (extracellular) of the rat α6 subunit linked to amino acids 233 to 499 (intracellular) of the rat α3 subunit. The previous study indicated that a novel TxIB from the *Conus textile*, whose sequence is GCCSDPPCRNKHPDLC-amide, was inferred from gene cloning [18]. Here, we showed the anti-addiction effect of α-conotoxin TxIB, a specific α6β2* nAChR antagonist, on nicotine-induced conditioned place preference (CPP). This would be new evidence to assure that the key role of α6β2* nAChR in nicotine addiction is recognized. 

## 2. Results

### 2.1. Structural Identification of α-Conotoxin TxIB

**Figure 1:**
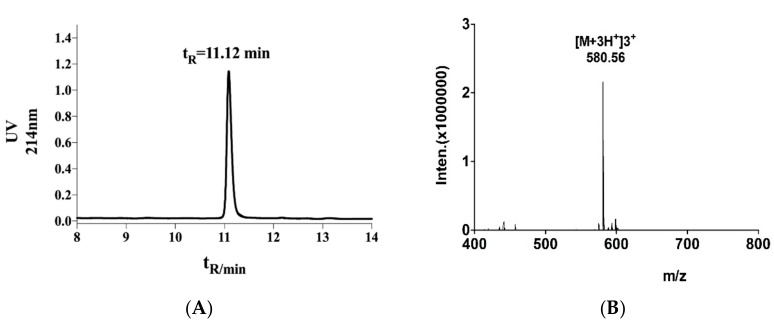
The HPLC profile (**A**) and the MS spectrometry (**B**) of α-conotoxin TxIB. HPLC and MS analysis of the TxIB synthesized by two-step oxidative folding. (**A**) is HPLC traces of TxIB. The time (t_R_) is the retention time of TxIB in HPLC profile. Analytical HPLC was carried out using an analytical Vydac C18 (5 μm, 4.6 mm × 250 mm). The separation method was as follows: a 10%–30% solvent B gradient over 30 min (solvent A is 0.1% TFA in H_2_O; solvent B is 0.05% TFA in 90% ACN). (**B**) is MS analysis of TxIB. Its calculated mass was 1738.68 Da.

The main peak was collected by high-performance liquid chromatography, and the exact molecular weight of α-conotoxin TxIB was measured by ESI-MS, the results are shown in Figure 1. The main absorption peak appeared at 11.12 min (A). By mass spectrometry, we could calculate the relative molecular mass of α-conotoxin TxIB to 1738.68 Da, which was consistent with the theoretical value of 1739.70 Da (B).

### 2.2. Nicotine-Induced Conditioned Place Preference in Mice 

**Figure 2:**
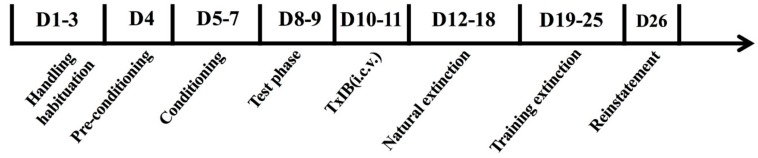
Schedule of conditioned place preference (CPP) experiments.

After three days of subcutaneous injection of nicotine (NIC) to establish a CPP model, the mice were subjected to intracerebroventricular surgery. Figure 2 was the schedule of the arrangement of the CPP experiment. It was commonly considered that mice with a CPP score ≥100 s were suitable for experiment. The overall ANOVA indicated that nicotine (0.5 mg/kg) produced a robust CPP score (F_2, 33_ = 38.57, *p* < 0.0001) (Table 1). From Table 1, we inferred that the NIC group was robust and stable by comparing the base value before/after CPP and after surgery. The animals of the control group spent equal time in drug-paired sides after saline injection compared with the initial average time. However, the average time that the NIC group spent in drug-paired sides was ~399.6 s, and was significantly longer than the base value before the CPP with ~236.3 s. 

### 2.3. Effect of Different Doses of TxIB on Locomotor Activity

**Figure 3:**
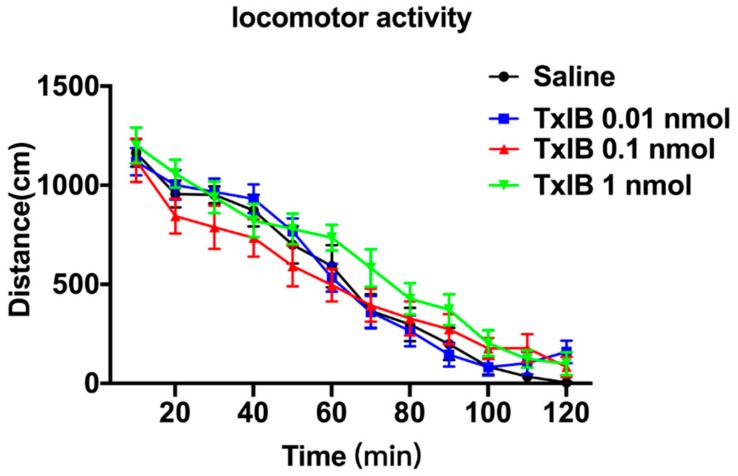
Effect of different doses of TxIB on locomotor activity in mice. The locomotor activity was recorded for 2 h. Data represent mean ± S.E.M. for 15 mice. There was no significance among the four groups.

The locomotor activity is shown in Figure 3. The ANOVA indicated that there was no significant difference in each dose of TxIB among each period compared with the saline group (F_33, 649_= 1.187, *p* = 0.22). Table 2 has shown the NIC-induced mice produced a notable total distance compared with the naïve mice. Following the injections of saline and TxIB, each dose produced little changes in total distance compared with the naïve group, and there was no significant difference between naïve group and TxIB group (*p* = 0.99). The results suggested that TxIB had no significant impact in naïve mice, nor in NIC-induced mice.

### 2.4. Effect of Different Doses of TxIB on Inhibition of CPP Expression

**Figure 4:**
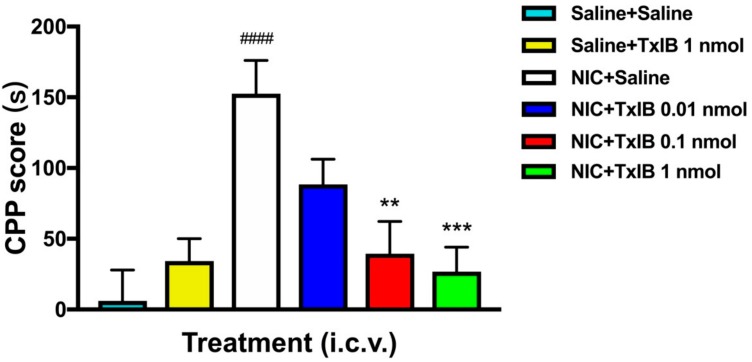
Effect of different doses of TxIB on NIC-induced CPP expression in mice. The CPP scores (s) were considered as the time spent in the drug-paired chamber after the injection of NIC/TxIB minus the initial time spent in the drug-paired chamber. Data represent mean ± S.E.M. for 10–12 mice (*n* = 10–12). # indicates significant difference from the Saline + Saline group; * indicates a significant difference from the NIC + Saline group (** = *p* < 0.01, *** = *p* < 0.001, #### = *p* < 0.001).

The effect of a single injection of α-conotoxin TxIB on NIC-induced CPP is shown in Figure 4. The overall ANOVA indicated that there was a significant effect of each dose of TxIB (F_5, 57_ = 7.257, *p* < 0.0001). Compared with the Saline + Saline group, the NIC + Saline group had a significant difference (*p* < 0.001), which indicated the success of the CPP model induced by NIC. In the beginning, all mice had no preference for any chamber in the CPP instrument, which was subsequently treated with saline or TxIB. The highest dose of TxIB with 1 nmol in the saline group itself did not produce a significant difference compared with Saline + Saline treated mice. No obvious effect of low-dose group of 0.01 nmol TxIB on CPP was observed. However, the NIC + TxIB high-dose (1 nmol) and medium-dose (0.1 nmol) groups inhibited CPP expression significantly compared with the NIC + Saline group (*p* < 0.01). As expected, α-conotoxin TxIB dose-dependently blocked NIC-induced CPP expression in mice.

### 2.5. Extinction of Nicotine-Induced CPP

**Figure 5:**
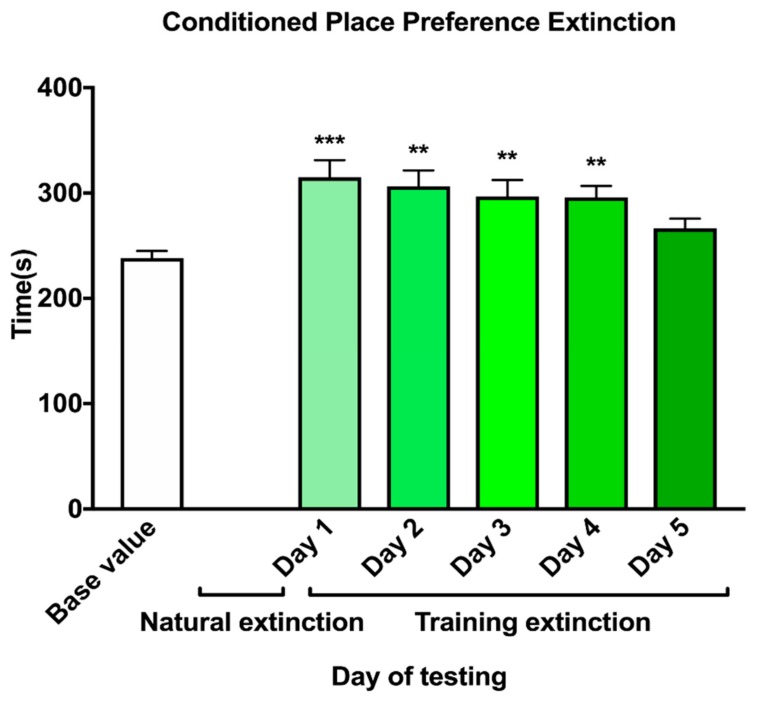
Extinction of NIC-induced CPP. Data represent mean ± S.E.M. The 250 base-value is essentially random entry into the chamber. Natural extinction lasted for 7 days without testing. Mice were extinguished on day 5 of the training extinction. Day 1–4 differed significantly when compared with the base value. ** indicates a significant difference in the NIC group compared with the base value (** = *p* < 0.01, *** = *p* < 0.001).

The result of the CPP extinction is shown in Figure 5. it indicated that all the animals exhibited a significant preference (F_5, 114_ = 5.002, *p* < 0.001) for the drug-paired side in 5 days. However, on day five, the mice displayed no significant preference compared to base value (*p* = 0.39). 

### 2.6. Effect of Different Doses of TxIB on Inhibition of CPP Reinstatement

**Figure 6:**
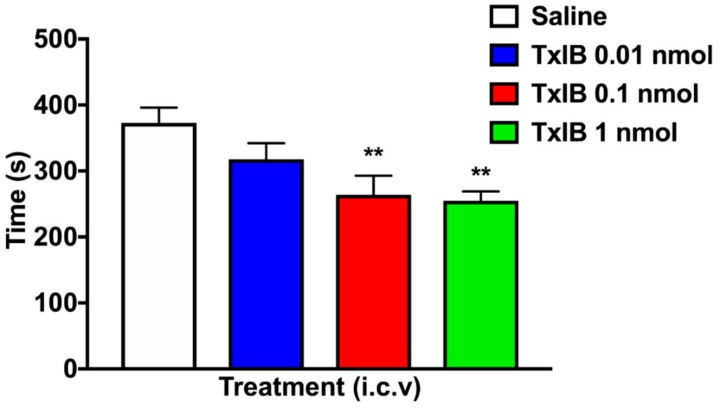
Effect of different doses of TxIB on inhibition of CPP reinstatement in mice. Data represent mean ± S.E.M. of 10 mice per group. TxIB could inhibit reinstatement in a dose-dependently manner. * indicates a significant difference in the TxIB + NIC group compared with the Saline + NIC group (** = *p* < 0.01).

After the progress of extinction, the mice no longer exhibited a significant preference. The result of reinstatement was shown in Figure 6. The overall ANOVA for reinstatement had a significant main effect for the various doses (F_3, 35_ = 5.645, *p* < 0.01). Compared with the Saline + NIC group, the time spent in the drug-paired chamber of the low dose (0.01 nmol), medium dose (0.1 nmol) and high dose (1 nmol) of TxIB had a significant decrease (*p* < 0.05). These data indicate that α-conotoxin TxIB successfully inhibited NIC-induced CPP reinstatement in mice with a dose-dependent manner.

### 2.7. Changes of Different Neurotransmitters in Several Brain Regions

**Figure 7:**
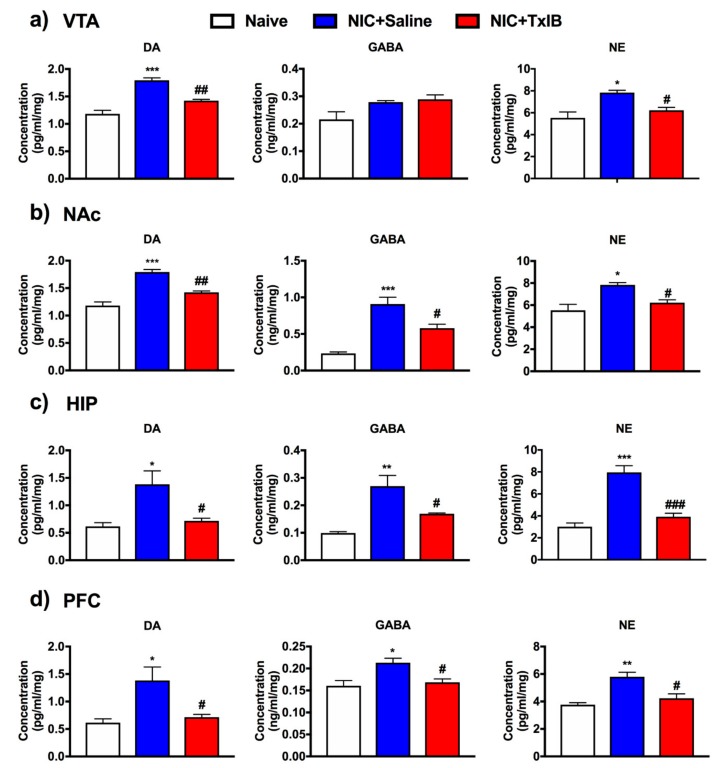
Changes of dopamine (DA), γ-aminobutyric acid (GABA) and noradrenaline (NE) in several brain regions. Data represent mean ± S.E.M. for 3–5 mice. TxIB may cause a decrease of DA, GABA and NE concentration in the nucleus accumbens (NAc), hippocampus (HIP) and prefrontal cortex (PFC) which was induced by nicotine. (**a**–**d**) represented changes of neurotransmitters in ventral tegmental area (VTA), NAc, HIP and PFC separately. In the VTA, the concentration of DA and NE were decreased, however, the concentration of GABA had few decreases. * represents a significant difference compared with the Naive group; # represents a significant difference compared to the NIC + Saline group (* = *p* < 0.05, ** = *p* < 0.01, *** = *p* < 0.001; # = *p* < 0.05, ## = *p* < 0.01, ### = *p* < 0.001).

To further explore how α-conotoxin TxIB acts on NIC-induced addiction, neurotransmitters in different brain regions were tested using ELISA (Figure 7). Statistical analysis is shown in Table 3. Compared with the naïve group, the concentration of DA in the nicotine group has increased in the VTA, NAc, hippocampus (HIP) and prefrontal cortex (PFC) respectively. As the β-hydroxylated derivative of DA, the concentration of noradrenaline (NE) has a corresponding increase, too (*p* < 0.05). Interestingly, the concentration of γ-aminobutyric acid (GABA) showed some differences. In nicotine mice, GABA has increased to a certain degree in NAc, HIP, and PFC. However, there was little increase in the VTA. After i.c.v. injection of the highest dose of 1 nmol TxIB, the concentration of DA and NE has significantly decreased compared with NIC + Saline group in VTA, while the concentration of GABA was not obvious (Figure 7a). In NAc, HIP and PFC, TxIB significantly prevented the concentration of these neurotransmitters (DA, GABA, NE) from rising sharply as it was shown in Figure 7b–d respectively. 

## 3. Discussion

Drug addiction is a chronically relapsing disorder, it is not a static phenomenon. Like other biobehavioral dysregulation, there are different components that constitute a circle of ever-growing pathology [19]. Positive reinforcement happens when the effect of the abused drug increases the possibility to obtain the drug again. The positive reinforcing effects of drugs is the pleasurable reward effects. The rewarding effect of a drug is the cause of the addiction process [20]. Conditioned place preference was used to assess the reward characteristics of nicotine in the present study. As it was previously mentioned, the mice injected with nicotine (0.5 mg/kg, s.c.) could induce a preference [21,22,23]. Consistent with previous studies, a robust CPP model was set up in our study. The results also approved that the mice had a stable CPP score after two days of surgery, indicating that this method was feasible and supported subsequent experiments. 

By studying the α6β2* nAChRs subtype and its neural structure in NIC-induced CPP model, α6β2* nAChRs could mediate the subjective reward effect and play a crucial role in CPP behavior study [24,25]. The α6β2* nAChRs were essential for self-administration of the nicotine in mice [26]. Currently, α-conotoxin MII, another 4/7 conotoxin, could potently block α6/α3β2β3 and α3β2 nAChRs. Whereas, a second-generation analog of α-conotoxin MII [H9A; L15A] was a much more potent at α6* than at α3* nAChRs [8]. The IC_50_ value at α6/α3β2β3 and α6β4 nAChRs is 2.4 and 270 nM, respectively [8]. MII [H9A; L15A] was examined in an animal experiment using the conditioned place preference paradigm [8,24].

In contrast, α-conotoxin TxIB is a novel peptide which can potently inhibit α6/α3β2β3 nAChR while having no obvious effect on other nAChR subtypes [18]. This is the advantage of TxIB compared with other conotoxins. The small peptide TxIB can bind the extracellular domain of the receptor, which is a transmembrane protein. So, TxIB does not need to reach the distant nuclei. TxIB was injected intracerebroventricularly, which should make it easy to diffuse into the extracellular spaces where the target α6β2* receptors are located.

Consistent with previous research [24], our behavioral studies further verified that α6β2* nAChRs play a critical role in rewarding effects and reinstatement of nicotine. TxIB blocked the expression of NIC-induced CPP, approving α6β2*-containing nAChRs play an important role in rewarding effects of nicotine. In addition, reinstatement was blocked by TxIB, proving that α6β2* nAChRs was involved in the mechanism of reinstatement. However, the pathway of reinstatement was unknown, it remains to be explored. Our animals experiment results showed that the dose of TxIB is higher than MII [H9A; L15A], the reason inferred may be the IC_50_ of MII [H9A; L15A] was lower.

In our study, the mice injected with nicotine were more active than the naïve mice, which was consistent with Benwell and Balfour et al. [27]. The reason could be that nicotine promoted the increase of dopamine transmission, and this could promote an increase in locomotor activity [27,28]. The observed effects of TxIB (i.c.v.) of each dose had no significant effect on locomotor activity, which was consistent with MII [H9A; L15A] [24]. This suggested that α6β2* nAChRs mediated the nicotine reward mechanism but could not regulate the locomotor activity. 

The Mesocorticolimbic (MCL) is thought to be associated with mood and nicotine addiction. Several neurotransmitters play a vital role in the rewarding and reinforcing effects of nicotine, especially dopamine [29]. Mice administrated with nicotine can increase dopaminergic neuron firing rates in the VTA [30], subsequently, dopamine was released rapidly in the NAc. This process was thought to explain the rewarding and reinforcing properties of nicotine [31,32]. The MCL such as VTA, NAc, PFC and HIP plays a crucial role in the reward system, especially NAc [33]. General chronic drug abuse, especially in high concentrations of nicotine-stimulated mice, leads to neuronal activation in the relevant brain regions, which shows significant changes and neuro-adaptability changes.

Nicotine passes through the blood–brain barrier with the flow of the blood in about 7 s and binds to related receptors in the brain after smoking. Whereafter, the release of various neurotransmitters (e.g., DA, GABA and NE) increased in the related brain regions. Nicotine binds to α6β2* nAChRs of dopaminergic neurons in midbrain, which can cause an increase of dopamine in NAc. Ultimately, the euphoria caused by dopamine leads to nicotine dependence [34]. The α6β2* nAChRs are mainly distributed in dopaminergic neurons of midbrain which were supported by immunoprecipitation and ligand binding studies [11,35,36,37,38,39]. TxIB could block the α6β2* nAChRs in dopaminergic neurons. Previous research has shown that α4β2 nAChR binding with nicotine was rapidly desensitized, and this inhibited the release of GABA due to DA neuronal afferents in VTA [34]. The concentration of GABA had no significant increase in VTA after nicotine addiction, it was the same as previous studies [34]. Our ELISA assay results displayed that the concentration of DA was increased after nicotine addiction, then TxIB (i.c.v.) inhibited the increase of dopamine in VTA and NAc. Repeated drug abuse is an associative learning process, which can alter neural plasticity in the HIP, VTA, PFC and NAc, playing an important role in the memory of relevant drug-seeking clues. Insufficiently, our research using ELISA has some limitations, and the neurotransmitters (DA, GABA and NE) levels are not representative of those in the extracellular space. Further research on the mechanism still needs to be explored.

The CPP model is based on Pavlov’s conditioning theory and combines rewards with learning and memory. The HIP is involved in learning and memory processes, and the PFC is associated with emotions. According to the mesocorticolimbic pathway, dopamine was projected from VTA to HIP and PFC. Our study shows that DA, NE and GABA were increased in HIP and PFC after nicotine addiction, then TxIB (i.c.v.) inhibited their increase.

In conclusion, α-conotoxin TxIB successfully inhibited NIC-induced CPP expression and reinstatement with a dose-dependent manner, and it had no significant effect on locomotor activity after intracerebroventricular injection of TxIB in mice. TxIB may prevent the binding ability of nicotine with α6β2* nAChRs on dopaminergic neurons, and DA, NE and GABA in some brain regions (VTA, NAc, HIP and PFC) were down-regulated. Furthermore, the results in this work indicated that α6β2* nAChRs played an important role in nicotine reward. This study demonstrates an excellent anti-addiction effect of TxIB on NIC-induced CPP, showing that the α6β2* nAChRs antagonist may offer a novel and effective route to develop new therapeutics for nicotine addiction. However, the mechanism of TxIB acted in the brain still requires further investigation, and the inability to penetrate the blood–brain barrier needs to be resolved. 

## 4. Materials and Methods

### 4.1. Chemical Synthesis of α-Conotoxin TxIB 

The linear peptides were synthesized by a solid-phase methodology using an ABI 433A peptide synthesizer by GL Biochem (Shanghai, China). Cys residues were protected in pairs either with S-trityl on Cys1 and Cys3, while S-acetamidomethyl on Cys2 and Cys4. The crude linear peptide was precipitated and purified by reversed-phase high-performance liquid chromatography (RP-HPLC, Waters). A two-step oxidation protocol was used to fold the peptides selectively as described previously [40]. The first step oxidative folding was carried out in an equal volume of potassium ferricyanide reaction buffer (20 mM K_3_[Fe(CN)_6_], 0.1 M Tris base; pH 7.5) to form the first disulfide bridge. The S-Acm protecting groups were removed by iodine oxidation for 5–10 min resulting in formation of the second disulfide bridge. The fully folded peptides were purified by HPLC on a reversed-phase Vydac C18 column. The sample was analyzed and identified by HPLC and mass spectrometry.

### 4.2. Animals

Male adult C57BL/6J mice (6 weeks of age, 20–23 g) were purchased from Hunan SJA Laboratory Animal Co., Ltd. (Changsha, China) with the permit SCXK 2016-0002, which were used for all the experiments. Animals were group-housed in the SPF animal raising room, Key Laboratory of Tropical Biological Resources, Ministry of Education, University of Hainan. Six mice were housed per cage and kept at a 23 ± 1 °C humidity-controlled environment and associated with a 12-h light/dark cycle light at 8 a.m. Experiments were performed during the light cycle. All the experiments were done in accordance with IASP guidelines on the use of awake animals with efforts made to minimize the number of animals and their discomfort [41].

### 4.3. Drugs

(-)-Nicotine hydrogen tartrate salt was purchased from Sigma-Aldrich Inc. (St. Louis, MO, USA). The dose of NIC was chosen based on previous studies [42]. NIC was dissolved in saline (0.9% sodium chloride) and was administered by subcutaneous injection in a volume of 10 mL/kg. α-conotoxin TxIB was administered by intracerebroventricular (i.c.v.) injection in a volume of 5 μL per mouse.

### 4.4. Intracerebroventricular Surgery

C57BL/6J mice were anesthetized with sodium pentobarbital (75 mg/kg, intraperitoneal injection, i.p.) on the morning after testing, a scalp incision was made to expose the bregma and prepare an injection site. A needle (O.D. 0.48 × I.D. 0.34 mm) with a sleeve tubing of polyurethane was used to make a hole in the skull at a site 0.6 mm AP, 1.3 mm ML, with respect to bregma at a depth of 2 mm. Mice were loosely sutured, leaving the injection site. The animals were allowed to recover from the anesthesia, 6 h later, the mice were woken up and housed one per cage with free access to water and standard laboratory food. A 26-gauge needle with a sleeve tubing of polyurethane was put into the lateral ventricle on the morning of testing. The needle was held in place for 20 s to ensure drug delivery. 

### 4.5. Nicotine-Induced Conditioned Place Preference (CPP) 

The CPP apparatus consisted of rectangular Plexiglas chambers divided into three distinct compartments with a PC computer equipped with an auto-monitoring system (AniLab, Ningbo, China). The left compartment had a bar grid floor and the front wall was black. The right compartment had a mesh floor and the front wall was white. The front wall of center compartment was gray (9.8 cm × 13.5 cm × 15 cm). Different floor textures in the black and white compartments (17.38 cm × 13.5 cm × 15 cm each) were to help the mice further differentiate between the two environments, and the middle compartment was neutral. The rewarding effects of nicotine were evaluated using the CPP paradigm, as previously described [43], modified to some extent.

Handling habituation: Mice came to our laboratory for three days of adaptation and had simple handling prior to the starting of the experiment. Each mouse was placed into the CPP apparatus for handling once per day for approximately 2 min. Notably, handling experience plays an important role in the ability of nicotine to produce CPP model [44].

Pre-conditioning phase: On the first day, each mouse was placed into the center chamber of the CPP apparatus, and allowed it access to explore the environment among the three chambers for 15 min. Time spent in each chamber was automatically recorded by the PC software and the initial preference for which side of the three chambers was determined. The mice which spent in one chamber for over 540 s were excluded from the study.

Conditioning phase: During this period, doors were closed so that animals cannot cross among the three chambers. Animals were confined in one side for conditioning approximately 20 min, with the control group received saline twice each day in both sides (morning at 8:00 and afternoon at 15:00) and NIC group received NIC (0.5 mg/kg, s.c.) in drug-paired sides and saline in the opposite side. Drug-paired sides were separated randomly among all the groups. In acquisition studies, conditioning lasted for 3 days, after each trial, the subjects returned to their own home cages [45]. 

Test phase: At 24 h after the last NIC treatment, the guillotine doors were removed and the animals had free access to the three chambers for 15 min. Time spent in each chamber was recorded. CPP score was expressed as time spent in the drug-paired side after the injection of NIC minus the initial time. A positive number indicated a preference for the drug-paired side, whereas a negative number indicated an aversion to the drug-paired side. A number at or near zero indicated no preference for either side. (i.e., an increase in preference for the drug-paired side ≥100 s compared with pre-conditioning test). The mice that meet the experimental requirements received intracerebroventricular surgery. In order to avoid the false positives of CPP, the test was performed twice in our study, including post-conditioning and after lateral ventricle catheterization surgery.

Extinction: Extinction period is a time duration in which NIC-induced CPP eliminates gradually. Natural extinction and training extinction were used for an extinguished CPP study. Natural extinction means that the mice were kept in their own home cages for one week after post-conditioning; Training extinction means that the mice were evaluated for preference every 24 h in a drug-free state until they had no significant preference compared with the pre-conditioning (time no more than ±20%) [46].

Reinstatement: Reinstatement means to seek the drug (nicotine) again after prolonged periods of abstinence. Exposure to the familiar environment, such as drug-paired chamber in CPP, as well as re-experience of drug effects, can cause the animals to seek the drug, and trigger reinstatement following extinction [47]. In the present study, on the reinstatement test day, mice were administrated TxIB or saline 90 min prior to the injection of NIC (0.1 mg/kg, s.c.). All the mice were allowed free access to the entire apparatus for 15 min. The time spent in each chamber was measured.

### 4.6. Treatment Protocol in Animals

On the day of the experiment, α-conotoxin TxIB was dissolved in 0.9% saline to reach the desired concentration, which was in a volume of 5 μL. To examine the effects of α-conotoxin TxIB on NIC-induced CPP, separate groups of animals were injected intracerebroventricularly with either saline or TxIB (0.01, 0.1, or 1 nmol) 90 min prior to placing them in the center compartment. The 90-min chosen was decided by the consideration of the state of the mice. The animals were anesthetized via inhalation with isoflurane and were injected intracerebroventricularly using an automatic drug delivery pump with Hamilton 1700, at a rate of 1 μL/min for a total of 5 μL. In the progress of reinstatement, the animals received pretreatment with TxIB 90 min prior to receiving a low dose NIC (0.1 mg/kg) injection.

### 4.7. Sample Preparation

Mice were sacrificed by cervical dislocation immediately after the post-conditioning test, then the brain was removed from the skull. Several brain regions such as midbrain VTA, NAc, HIP and PFC were quickly dissected on an ice-cold metal plate and were weighed. liquid nitrogen was added and ground thoroughly, then samples were homogenized in an ice-cold PBS buffer using a grinding rod and thoroughly ground. The particulate fraction was obtained by centrifugation at 12,000 rpm, 4 °C for 10 min in a himac CF 16RX Versatile Compact Centrifuge. Each sample was taken from the supernatant and stored as a pellet under a homogenization buffer at −80 °C until use. 

### 4.8. Enzyme-Linked Immunosorbent Assay

The concentration of the sample was measured with the double antibody sandwich method. The sample was taken out from −80 °C and slowly thawed on ice. Blank well, standard wells and sample wells were set on the enzyme-labeled plates. The standard sample was accurately loaded with 50 µL. The sample dilution was first added in sample wells with 40 µL, then a 10 µL sample was added to be tested (the final dilution of the sample is 5 times). The plate was sealed with microplate sealers and was incubated at 37 °C for 30 min. The washing solution was used to wash the walls with five times, 50 µL of horseradish peroxidase (HRP)-labeled dopamine antibody was added and incubated for another 30 min to form an antibody-antigen-enzyme-labeled antibody complex. After thorough washing again, the substrate was stained with TMB. Absorbance was read at 450 nm on a Spectramax microplate reader (Molecular Devices Corp., SpectraMax M2, 06176, Downingtown, PA, USA). A blank was subtracted from all other absorbance. The competitive curves were analyzed with a four-parameter logistic equation and concentration was calculated from the standard curve and expressed as concentration/weight.

### 4.9. Locomotor Activity

The locomotor activity experiment was performed to assess whether TxIB has agonistic or sedative effects in C57BL/6J mice. The mice were placed into individual activity cages (40 × 40 × 35 cm; AniLab, Ningbo, China) and handled for 5 min to habituate to the apparatus before starting the experiments, once per day for three days. On the test day, the mice were injected with α-conotoxin TxIB/saline respectively and were placed in the test chambers where counts of locomotor activity were recorded for 120 min.

In order to further assess the influence of TxIB in NIC-induced mice, we fetched the total distance in 15 min on the CPP test day. During the CPP test, the total distance of the animals following drug and vehicle injections was recorded on all days. These data were captured by the auto-monitoring system (AniLab, Ningbo, China), which tracks and records all the movements/positional changes of the animals.

### 4.10. Statistical Analysis

All data were expressed as mean ± S.E.M. and analyzed using Graphpad Prism (version 7 for Windows(TM), San Diego, CA, USA). For the CPP expression, extinction, reinstatement experiment and ELISA assay, these data were analyzed by one-way ANOVA and post hoc Bonferroni’s multiple comparison analyzes. For the total distance in the CPP model, the nonparametric test and Kruskal–Wallis multiple comparisons were used to analyze. As for locomotor activity experiment, two-way ANOVA was performed with Bonferroni’s multiple comparisons test. The level of significance was set at *p* < 0.05.

## Figures and Tables

**Table 1:** Time spent in the drug-paired chamber of nicotine (NIC) induced CPP in mice (s).

Group	Base Value before CPP	Base Value after CPP	Base Value after Surgery
Saline	246.2 ± 7.881	252 ± 9.766	272.1 ± 17.06
NIC	236.3 ± 9.353	399.6 ± 16.83 ****	399.6 ± 17.91 ****

The above table suggested that the CPP model (0.5 mg/kg) was robust and stable after intracerebroventricular surgery. * indicates a significant difference in the NIC group compared with the Saline group (**** = *p* < 0.0001).

**Table 2:** The total distance in the CPP model.

Treatment Group	Total Distance in Drug-Paired Compartment
Naive	730.8 ± 21.19
NIC	939 ± 42.86 *
NIC + Saline	661.7 ± 47.47
NIC + TxIB 0.01 nmol	626.2 ± 82
NIC + TxIB 0.1 nmol	704.9 ± 46.57
NIC + TxIB 1 nmol	683 ± 65.21

Mice were treated with saline or TxIB (0.01–1 nmol, i.c.v.) for the CPP assessment on test day. The total distance was taken in the chamber of 15 min (mean ± S.E.M., totally for at least 10 mice). * indicates a significant difference compared with the naïve mice (* = *p* < 0.05).

**Table 3:** Effect of nicotine (0.5 mg/kg) administration on neurotransmitters levels in the nucleus accumbens, ventral tegmental area, hippocampus and prefrontal cortex of saline or TxIB pretreatment. Statistical analysis was performed using one-way ANOVA, followed by post hoc comparisons using the Bonferroni test.

	Neurotransmitters	DA		GABA		NE	
Brain Regions		F Value	*p* Value	F Value	*p* Value	F Value	*p* Value
VTA		F_(2, 6)_ = 42.05	<0.001	F_(2, 8)_ = 5.074	<0.05	F_(2, 6)_ = 10.25	<0.05
NAc		F_(2, 6)_ = 42.05	<0.001	F_(2, 7)_ = 22.17	<0.001	F_(2, 6)_ = 10.25	<0.05
HIP		F_(2, 6)_ = 7.267	<0.05	F_(2, 9)_ = 14.18	<0.01	F_(2, 9)_ = 36.01	<0.0001
PFC		F_(2, 7)_ = 9.148	<0.05	F_(2, 7)_ = 8.468	<0.05	F_(2, 8)_ = 12.25	<0.01

## Abbreviations

nAChR: Nicotinic acetylcholine receptor; CPP: Conditioned place preference; ELISA: Enzyme linked immunosorbent assay; DA: Dopamine; GABA: γ-aminobutyric acid; NE: Noradrenaline; VTA: Ventral tegmental area; NAc: Nucleus accumbens; PFC: Prefrontal cortex; HIP: Hippocampus; CNS: Central nervous system.
